# Supplementary material for: Daily-Life Walking Speed, Quality and Quantity Derived from a Wrist Motion Sensor: Large-Scale Normative Data for Middle-Aged and Older Adults
Source: Sensors (Basel). 2024 Aug 10;24(16):5159. doi: 10.3390/s24165159 (PMC11359895; doi:10.3390/s24165159)
Supplement: Supplementary file 1 [file sensors-24-05159-s001.zip › sensors-3056898-supplementary.pdf]

Supplementary material

Daily-Life Walking Speed, Quality and Quantity Derived from a Wrist Motion Sensor: Large-Scale Normative Data For Middle-Aged and Older Adults

Lloyd L. Y. Chan [1,2], Stephen R. Lord [1,3] and Matthew A. Brodie [4,*]

[1] Neuroscience Research Australia, Sydney, New South Wales 2031, Australia; l.chan@neura.edu.au (L.L.Y.C.); s.lord@neura.edu.au (S.R.L.)
[2] School of Health Sciences, University of New South Wales, Sydney, New South Wales 2052, Australia
[3] School of Population Health, University of New South Wales, Sydney, New South Wales 2052, Australia
[4] Graduate School of Biomedical Engineering, University of New South Wales, Sydney, New South Wales 2052, Australia
* Correspondence: a.m.brodie@unsw.edu.au

Supplementary Table S1. The mean and standard deviation, or median and interquartile range of digital sleep and gait biomarkers, based on a minimum wear time of three days, each lasting at least twelve hours.

	Sex		Age group				
**Digital sleep and gait biomarkers, units**	Female (N=51766)	Male (N=40110)	45 to 54 (N=18699)	55 to 64 (N=31561)	65 to 74 (N=38728)	75 to 79 (N=2888)	Total (N=91876)
**Sleep**							
Sleep duration, hour†	8.0 (1.05)	8.3 (1.24)	7.9 (1.08)	8.1 (1.12)	8.3 (1.17)	8.3 (1.21)	8.1 (1.15)
Bedtime, hour of the day†	23:00 (1hr17min)	22:54 (1hr25min)	22:48 (1hr32min)	23:00 (1hr22min)	23:06 (1hr14min)	23:06 (1hr12min)	23:00 (1hr21min)
**Gait Quantity**							
Steps per day†	7748 (2694)	7290 (2648)	7943 (2641)	7818 (2712)	7226 (2624)	6363 (2566)	7548 (2683)
**Walk Length distribution**							
Longest continuous walk duration, second‡	324.6 [185, 529]	331.4 [191, 546]	346.9 [208, 554]	347.4 [198, 564]	308.7 [175, 513]	252.0 [135, 446]	327.4 [187, 537]
% Walk ≥ 8 second†	37.7 (5.21)	40.3 (5.78)	40.3(5.2)	39.4 (5.5)	37.9 (5.7)	36.3 (6.0)	38.9 (5.61)
% Walk ≥ 60 second‡	1.5 [0.8, 2.6]	1.9 [1.0, 3.3]	1.9 [1.0, 3.1]	1.8 [0.9, 3.0]	1.5 [0.8, 2.7]	1.3 [0.6, 2.5]	1.7 [0.9, 2.9]
**Gait Speed and Intensity**							
Maximal walking speed, centimetre per second†	145.3 (6.80)	153.8 (7.61)	152.0 (7.83)	149.8 (8.02)	147.3 (8.18)	144.9 (8.28)	149.0 (8.29)
Usual walking speed, centimetre per second†	111.1 (6.61)	118.4 (7.60)	117.5 (7.48)	115.1 (7.66)	112.4 (7.75)	110.2 (7.77)	114.3 (7.94)
Cadence median, spm †	101.6 (3.16)	100.1 (3.04)	101.2 (3.13)	101.1 (3.16)	100.7 (3.21)	100.6 (3.48)	100.9 (3.19)
**Gait Quality**							
Step-time variability, millisecond†	57.9 (12.10)	64.8 (15.18)	59.3 (13.13)	60.1 (13.72)	62.1 (14.34)	63.5 (14.90)	60.9 (13.96)
Step regularity, %†	65.5 (4.76)	65.2 (4.77)	66.0 (4.42)	65.7 (4.63)	64.9 (4.92)	64.1 (5.27)	65.4 (4.76)
Stride regularity, %†	65.5 (4.76)	65.2 (4.77)	52.8 (4.45)	52.6 (4.54)	52.1 (4.72)	51.6 (4.90)	52.4 (4.62)
**Walk hand positions**							

% Walk with Arm Swings‡	91.7 [86.5, 95.3]	93.4 [89.2, 96.2]	91.7 [86.6, 95.2]	92.3 [87.1, 95.6]	93.1 [88.4, 96.1]	93.4 [89.0, 96.4]	92.5 [87.6, 95.8]
% Texting‡	0.5 [0.3, 0.8]	0.4 [0.2, 0.7]	0.4 [0.3, 0.7]	0.4 [0.3, 0.8]	0.5 [0.3, 0.8]	0.5 [0.3, 0.9]	0.4 [0.3, 0.8]
% Phonecall‡	0.4 [0.1, 0.9]	0.3 [0.1, 0.8]	0.4 [0.1, 0.9]	0.4 [0.1, 0.9]	0.4 [0.1, 0.8]	0.4 [0.1, 1.0]	0.4 [0.1, 0.9]
% Hands in pockets‡	1.0 [0.5, 1.7]	0.8 [0.5, 1.5]	1.2 [0.7, 2.1]	1.0 [0.6, 1.7]	0.8 [0.4, 1.3]	0.7 [0.4, 1.1]	0.9 [0.5, 1.6]
% Shoulder bag‡	3.6 [1.8, 6.6]	2.9 [1.5, 5.5]	3.7 [1.9, 6.6]	3.5 [1.8, 6.4]	3.1 [1.5, 5.7]	2.8 [1.4, 5.4]	3.3 [1.7, 6.1]
% Briefcase‡	1.6 [0.8, 3.0]	0.9 [0.5, 1.7]	1.4 [0.7, 2.6]	1.3 [0.6, 2.5]	1.1 [0.6, 2.3]	1.1 [0.5, 2.2]	1.2 [0.6, 2.4]
% Static which includes texting and cellphone‡	1.0 [0.5, 1.8]	0.8 [0.4, 1.5]	0.9 [0.5, 1.6]	0.9 [0.5, 1.7]	0.9 [0.5, 1.7]	1.0 [0.5, 1.9]	0.9 [0.5, 1.7]
% Adaptive which includes shoulder bag, brief case and hands in pockets‡	6.9 [3.8, 11.6]	5.4 [3.0, 9.2]	7.0 [4.0, 11.8]	6.5 [3.6, 11.1]	5.7 [3.1, 9.8]	5.2 [2.7, 8.9]	6.2 [3.4, 10.6]

† indicates that the variable is presented as mean (standard deviation). ‡ indicates that the variable is presented as median [interquartile range].

Supplementary Table S2. Reference values (percentiles) for women in each digital gait biomarker for age groups 45-54, 55-64, 65-74 and 75-79.

Digital sleep and gait biomarkers, units	Age Group	5%	10%	25%	50%	75%	90%	95%
**Sleep**								
Sleep duration, hour	45-54	6.9	7.1	7.6	8.1	8.7	9.3	9.7
	55-64	6.9	7.2	7.7	8.2	8.8	9.5	9.9
	65-74	6.9	7.2	7.7	8.3	8.9	9.6	10.1
	75-79	6.8	7.2	7.7	8.2	8.9	9.6	10.1
Bedtime, hour of the day	45-54	21.7	22.1	22.6	23.2	23.8	24.5	24.9
	55-64	21.7	22.1	22.7	23.3	23.9	24.6	25
	65-74	21.7	22.1	22.8	23.3	24.0	24.6	25.1
	75-79	21.6	22.1	22.7	23.4	24.1	24.6	25.1
**Gait Quantity**								
Steps per day	45-54	4511	5222	6507	8084	9945	11901	13264
	55-64	4273	4994	6281	7898	9821	11771	13103
	65-74	3699	4399	5689	7304	9183	11103	12371
	75-79	2808	3245	4547	6247	7920	9748	11359
Longest continuous walk duration, second	45-54	93.3	124.0	206.9	356.0	568.0	828.9	1016.1
	55-64	86.6	118.7	198.3	353.1	568.0	836.0	1033.7
	65-74	70.0	98.9	169.3	301.6	500.0	744.6	940
	75-79	45.5	64.5	119.2	216.7	415.4	636.0	820.3
**Walk Length Distribution**								
% Walk ≥ 8 second	45-54	31.6	33.2	36.1	39.2	42.3	45.2	47.1
	55-64	30.0	32.0	35.1	38.3	41.5	44.6	46.4
	65-74	28.3	30.2	33.4	36.6	39.9	43.0	45
	75-79	24.6	26.8	30.6	34.6	38.2	41.4	43.4
% Walk ≥ 60 second	45-54	0.3	0.4	0.9	1.7	2.8	4.3	5.3
	55-64	0.2	0.4	0.8	1.6	2.7	4.1	5.1
	65-74	0.1	0.3	0.7	1.3	2.4	3.6	4.5
	75-79	0.0	0.1	0.4	1.0	2.0	3.4	4.3
**Gait Speed and Intensity**								
Maximal walking speed, centimetre per second	45-54	138.9	140.9	144.4	148.1	152.2	156.3	158.9
	55-64	136.3	138.4	142.0	146.0	150.2	154.4	157.2
	65-74	133.0	135.4	139.0	142.9	147.1	151.3	154
	75-79	130.1	132.3	135.8	140.1	144.4	148.0	150.1
Usual walking speed, centimetre per second	45-54	104.9	106.8	110.2	113.9	118.1	122.1	124.8
	55-64	102.2	104.3	107.8	111.7	115.8	120.1	122.9
	65-74	99.2	101.3	104.8	108.5	112.6	116.8	119.5
	75-79	96.7	98.6	101.9	105.8	109.9	114.2	116.9
Cadence median, spm	45-54	97.9	98.7	99.9	101.3	102.9	105.1	106.8
	55-64	97.7	98.6	99.8	101.2	102.9	105.1	106.9
	65-74	97.3	98.2	99.6	101.0	102.7	105.0	106.8
	75-79	97.1	98.3	99.5	100.9	102.6	104.6	106.7
**Gait Quality**								
Step-time variability, millisecond	45-54	41.2	44.4	49.7	55.7	62.9	71.1	76.5
	55-64	41.1	44.4	49.8	56.4	63.9	72.4	78.1

	65-74	41.1	44.7	50.8	57.7	65.9	74.2	79.7
	75-79	41.2	45.6	51.7	59.9	67.1	75.4	82.4
Step regularity, %	45-54	59.1	60.8	63.4	66.2	68.8	71.2	72.6
	55-64	58.5	60.3	63.2	66.0	68.8	71.2	72.6
	65-74	56.7	58.8	62.0	65.1	68.1	70.6	72.3
	75-79	54.1	57.1	60.7	64.0	67.2	69.8	71.4
Stride regularity, %	45-54	46.3	47.6	49.8	52.4	55.2	57.7	59.5
	55-64	45.9	47.3	49.7	52.3	55.0	57.7	59.4
	65-74	44.9	46.5	48.9	51.6	54.5	57.5	59.3
	75-79	43.4	45.3	48.1	50.9	54.0	56.8	58.4
**Walk Hand Positions**								
% Walk with Arm Swings	45-54	73.4	78.1	85.3	90.8	94.7	96.9	97.9
	55-64	74.5	79.4	86.2	91.6	95.2	97.3	98.1
	65-74	76.8	81.4	87.5	92.6	95.9	97.8	98.6
	75-79	77.1	82.3	88.9	93.2	96.5	98.0	98.8
% Texting	45-54	0.1	0.2	0.3	0.5	0.8	1.2	1.6
	55-64	0.1	0.2	0.3	0.5	0.8	1.3	1.7
	65-74	0.1	0.1	0.3	0.5	0.8	1.3	1.8
	75-79	0.1	0.1	0.3	0.5	0.9	1.4	1.8
% Phonecall	45-54	0.0	0.0	0.1	0.4	0.9	1.9	2.8
	55-64	0.0	0.0	0.1	0.4	0.9	1.9	2.8
	65-74	0.0	0.0	0.1	0.4	0.9	1.9	2.9
	75-79	0.0	0.0	0.1	0.4	1.1	2.2	3.6
% Hands in pockets	45-54	0.3	0.4	0.7	1.2	2.2	3.8	5.1
	55-64	0.2	0.3	0.6	1.0	1.8	3.1	4.3
	65-74	0.2	0.2	0.4	0.8	1.4	2.3	3.2
	75-79	0.1	0.2	0.3	0.6	1.1	1.7	2.5
% Shoulder bag	45-54	0.7	1.1	2.1	4.1	7.2	11.5	14.9
	55-64	0.6	0.9	1.9	3.7	6.7	11.0	14.1
	65-74	0.4	0.7	1.5	3.2	6.0	9.9	12.8
	75-79	0.4	0.6	1.3	2.9	5.3	9.1	11
% Briefcase	45-54	0.3	0.5	0.9	1.7	3.2	5.3	7.1
	55-64	0.2	0.4	0.8	1.6	3.1	5.2	7.2
	65-74	0.2	0.3	0.7	1.5	2.9	5.0	6.7
	75-79	0.1	0.3	0.6	1.3	2.6	4.6	6.3
% Static which includes texting and cellphone	45-54	0.2	0.3	0.5	1.0	1.8	2.9	4
	55-64	0.2	0.3	0.5	1.0	1.8	3.0	4.1
	65-74	0.2	0.3	0.5	0.9	1.8	3.1	4.2
	75-79	0.1	0.3	0.5	1.0	2.0	3.5	5.1
% Adaptive which includes shoulder bag, brief case and hands in pockets	45-54	1.7	2.5	4.4	7.9	12.9	19.3	24
	55-64	1.4	2.1	3.9	7.1	12.0	18.1	22.7
	65-74	1.1	1.7	3.3	6.2	10.6	16.0	20.1
	75-79	0.9	1.4	2.7	5.5	9.0	14.3	18.6

Supplementary Table S3. Reference values (percentiles) for men in each digital gait biomarker for age groups 45-54, 55-64, 65-74 and 75-79.

Digital sleep and gait biomarkers, units	Age Group	5%	10%	25%	50%	75%	90%	95%
**Sleep**								
Sleep duration, hour	45-54	6.7	7.0	7.5	8.1	8.7	9.4	10.0
	55-64	6.9	7.2	7.7	8.3	9.0	9.8	10.4
	65-74	7.0	7.3	7.9	8.5	9.3	10.2	10.9
	75-79	6.8	7.2	7.8	8.5	9.3	10.2	10.8
Bedtime, hour of the day	45-54	21.4	21.9	22.6	23.2	23.9	24.6	25.1
	55-64	21.3	21.8	22.5	23.2	23.9	24.5	25.0
	65-74	21.3	21.8	22.6	23.2	23.9	24.5	25.0
	75-79	21.3	21.8	22.5	23.2	23.9	24.6	25.0
**Gait Quantity**								
Steps per day	45-54	4261	4917	6142	7630	9427	11326	12686
	55-64	3917	4620	5881	7463	9341	11342	12723
	65-74	3336	4051	5277	6825	8611	10535	11880
	75-79	2770	3359	4578	6053	7714	9630	11244
Longest continuous walk duration, second								
	45-54	104.8	138.0	214.0	352.8	565.7	836.5	1071.8
	55-64	92.7	121.7	201.3	356.6	583.2	873.4	1091.8
	65-74	75.4	105.3	178.9	319.3	538.4	824.0	1051.3
**Walk Length Distribution**	75-79	60.9	87.1	145.1	271.3	480.0	746.9	918.6
% Walk ≥ 8 second	45-54	33.8	35.7	38.6	42.1	45.3	48.7	50.7
	55-64	32.2	34.2	37.5	41.0	44.6	48.0	50.3
	65-74	29.8	32.1	35.6	39.2	43.1	46.5	48.7
	75-79	28.0	30.4	34.0	38.0	42.0	45.1	47.6
% Walk ≥ 60 second	45-54	0.4	0.6	1.2	2.1	3.4	5.1	6.3
	55-64	0.3	0.5	1	2	3.4	5.1	6.3
	65-74	0.2	0.4	0.9	1.7	3.1	4.8	6.1
	75-79	0.1	0.3	0.7	1.6	2.9	4.5	5.9
**Gait Speed and Intensity**								
Maximal walking speed, centimetre per second	45-54	147.1	149.3	152.8	157.0	161.6	166.3	169.4
	55-64	144.0	146.3	150.2	154.7	159.3	164.1	167.6
	65-74	140.3	142.8	146.9	151.5	156.4	161.1	164.2
	75-79	136.6	139.5	143.9	148.5	153.1	158.7	162.8
Usual walking speed, centimetre per second	45-54	112.0	114.1	117.7	121.7	126.4	131.2	134.6
	55-64	108.7	111.0	114.8	119.2	124.0	128.7	132.2
	65-74	105.1	107.5	111.3	115.8	120.7	125.8	129.3
	75-79	101.9	104.5	108.7	112.9	117.7	122.6	125.8
Cadence median, spm	45-54	96.5	97.4	98.7	100.1	101.5	103.1	104.4
	55-64	96.0	97.0	98.5	100.0	101.5	103.2	104.7
	65-74	95.5	96.5	98.2	99.8	101.3	103.1	104.7
	75-79	95.2	96.3	98.0	99.8	101.5	103.6	105.5
**Gait Quality**								
Step-time variability, millisecond	45-54	44.1	47.7	53.8	61.3	71.0	81.4	89.2
	55-64	43.7	47.7	54.3	62.5	72.8	83.8	91.5

	65-74	44.0	48.5	55.5	64.3	74.5	85.8	93.8
	75-79	44.5	48.7	56.3	65.4	75.8	87.1	94.1
Step regularity	45-54	59.4	60.9	63.3	65.9	68.4	70.8	72.3
	55-64	58.4	60.1	62.9	65.7	68.3	70.7	72.2
	65-74	56.5	58.8	62.0	65.0	67.9	70.5	72.1
	75-79	54.9	57.4	61.3	64.5	67.5	70.2	71.9
Stride regularity	45-54	47.3	48.6	50.6	52.8	55.2	57.7	59.4
	55-64	46.7	48.1	50.3	52.6	55.1	57.7	59.3
	65-74	45.8	47.4	49.8	52.2	54.8	57.5	59.4
	75-79	44.6	46.5	49.2	51.8	54.5	57.0	59.4
**Walk Hand Positions**								
% Walk with Arm Swings	45-54	78.2	83.0	88.8	93.1	95.8	97.5	98.2
	55-64	78.5	83.1	89.0	93.4	96.1	97.7	98.4
	65-74	79.6	83.9	89.5	93.7	96.4	97.9	98.5
	75-79	79.4	83.8	89.3	93.7	96.5	97.9	98.5
% Texting	45-54	0.1	0.1	0.2	0.4	0.6	1.0	1.4
	55-64	0.1	0.1	0.2	0.4	0.7	1.1	1.5
	65-74	0.1	0.1	0.2	0.4	0.7	1.2	1.6
	75-79	0.1	0.1	0.3	0.5	0.8	1.4	2.0
% Phonecall	45-54	0	0	0.1	0.3	0.7	1.4	2.0
	55-64	0	0	0.1	0.3	0.8	1.5	2.2
	65-74	0	0	0.1	0.3	0.8	1.5	2.4
	75-79	0	0	0.1	0.4	0.9	1.9	3.2
% Hands in pockets	45-54	0.3	0.4	0.6	1.1	2.0	3.6	5.1
	55-64	0.2	0.3	0.5	0.9	1.6	2.9	4.3
	65-74	0.2	0.2	0.4	0.7	1.3	2.3	3.3
	75-79	0.1	0.2	0.4	0.7	1.2	2.1	3.1
% Shoulder bag	45-54	0.5	0.8	1.6	3.1	5.6	9.6	12.9
	55-64	0.5	0.8	1.6	3.0	5.7	9.9	13.7
	65-74	0.4	0.7	1.4	2.8	5.3	9.5	13.1
	75-79	0.4	0.7	1.3	2.7	5.4	8.9	12.9
% Briefcase	45-54	0.2	0.3	0.5	0.9	1.7	3.2	4.5
	55-64	0.2	0.2	0.5	0.9	1.7	3.1	4.3
	65-74	0.2	0.2	0.5	0.9	1.7	3.1	4.3
	75-79	0.1	0.2	0.5	0.9	1.7	3.2	4.8
% Static which includes texting and cellphone	45-54	0.2	0.2	0.4	0.8	1.4	2.3	3.1
	55-64	0.2	0.2	0.4	0.8	1.5	2.5	3.4
	65-74	0.2	0.2	0.4	0.8	1.5	2.6	3.7
	75-79	0.2	0.3	0.5	0.9	1.8	3.4	4.7
% Adaptive which includes shoulder bag, brief case and hands in pockets	45-54	1.4	2.0	3.4	5.9	9.7	15.3	19.8
	55-64	1.2	1.8	3.1	5.5	9.5	15	19.3
	65-74	1.1	1.6	2.8	5.1	8.8	14	17.9
	75-79	1.0	1.6	2.7	5.0	8.8	13.6	17.6

Supplementary Table S4. Mean absolute percentage error (MAPE) of sensor-based step time and walking speed estimations.

**Hand positions while walking**	**Mean absolute percentage error in step time ±SD(%)**	**Mean absolute percentage error in walking speed ± SD(%)**
Arm-Swing	5.2 ± 11.2	5.7 ± 8.5
Hands in pocket	3.8 ± 13.1	-
Texting	2.9 ± 4.3	-
Phone-call	2.8 ± 5.9	-
Shoulder bag	2.9 ± 6.7	-
Briefcase	3.3 ± 8.9	-

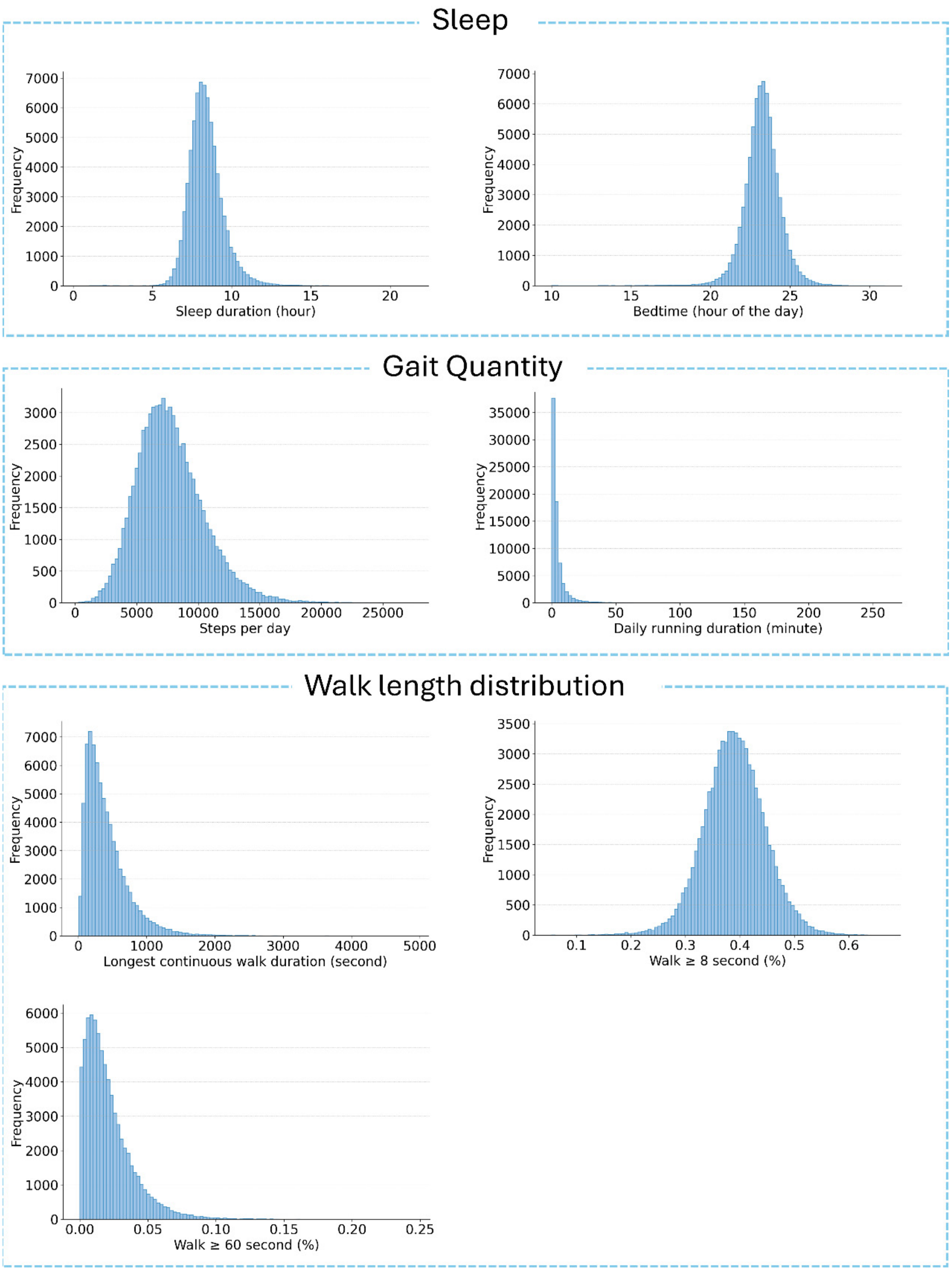

Supplementary Figure S1. Histograms illustrating the distribution of sleep, gait quantity and distribution biomarkers.

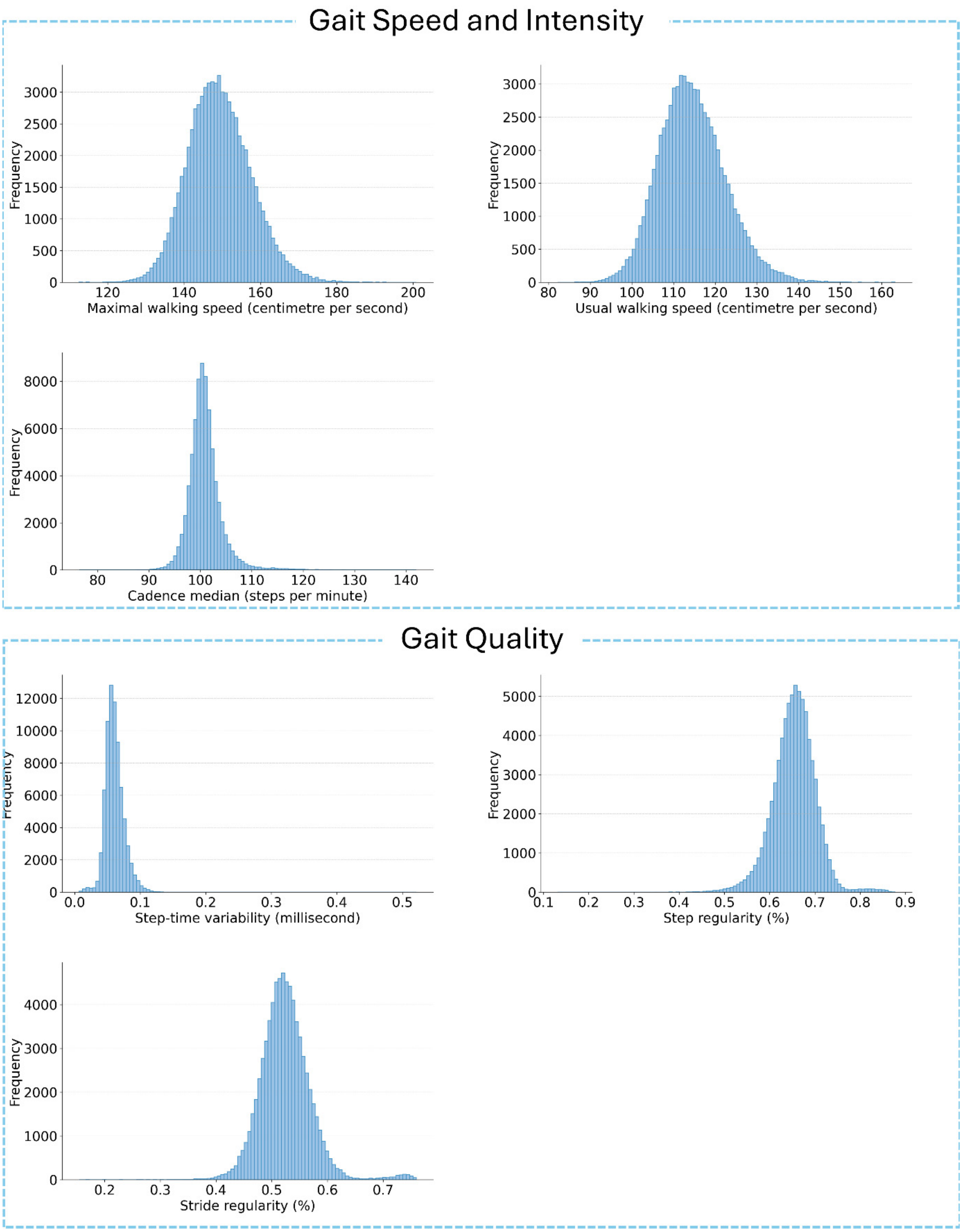

Supplementary Figure S2. Histograms illustrating the distribution of gait speed, intensity and quality biomarkers.

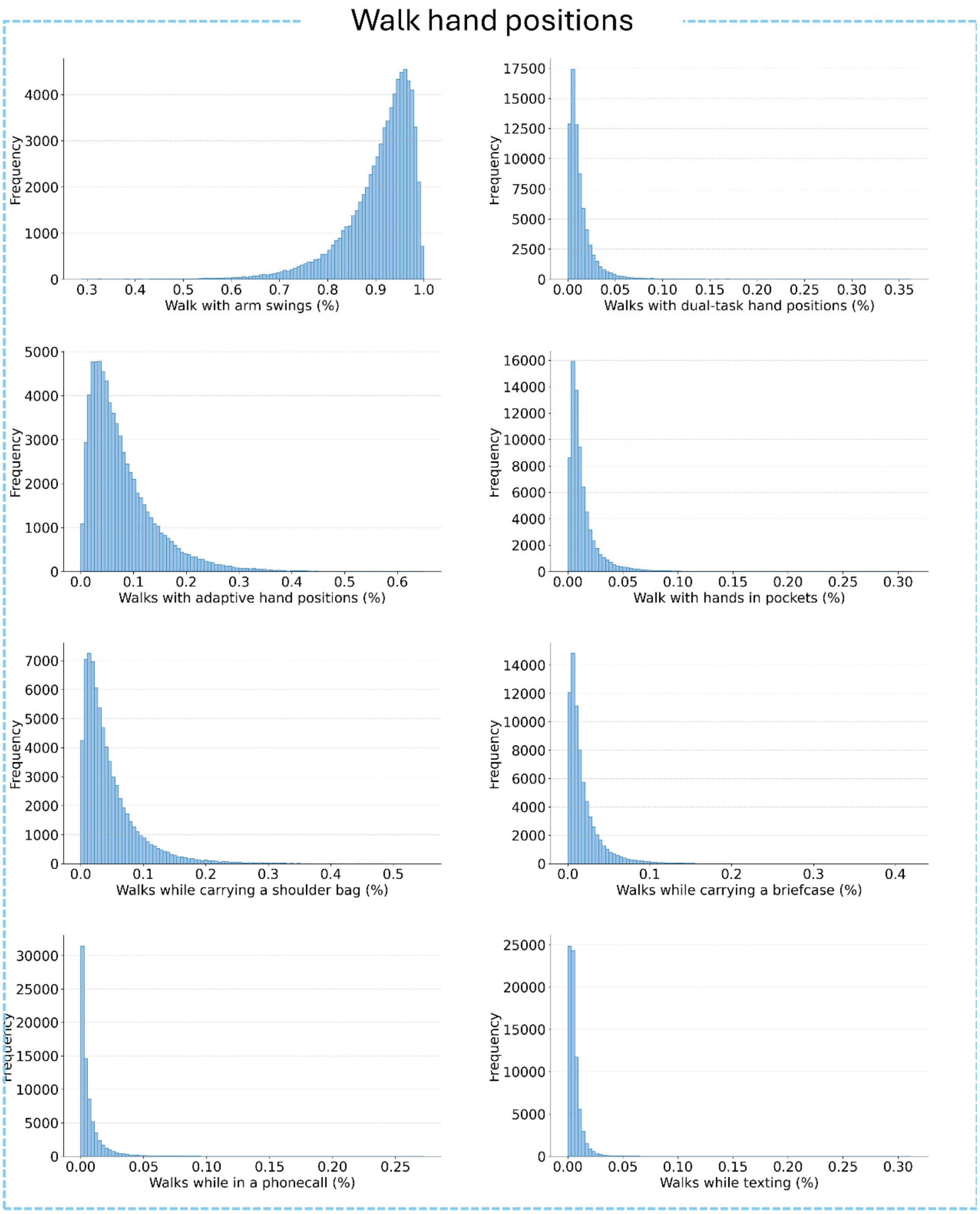

Supplementary Figure S3. Histograms illustrating the distribution of walk hand position biomarkers.

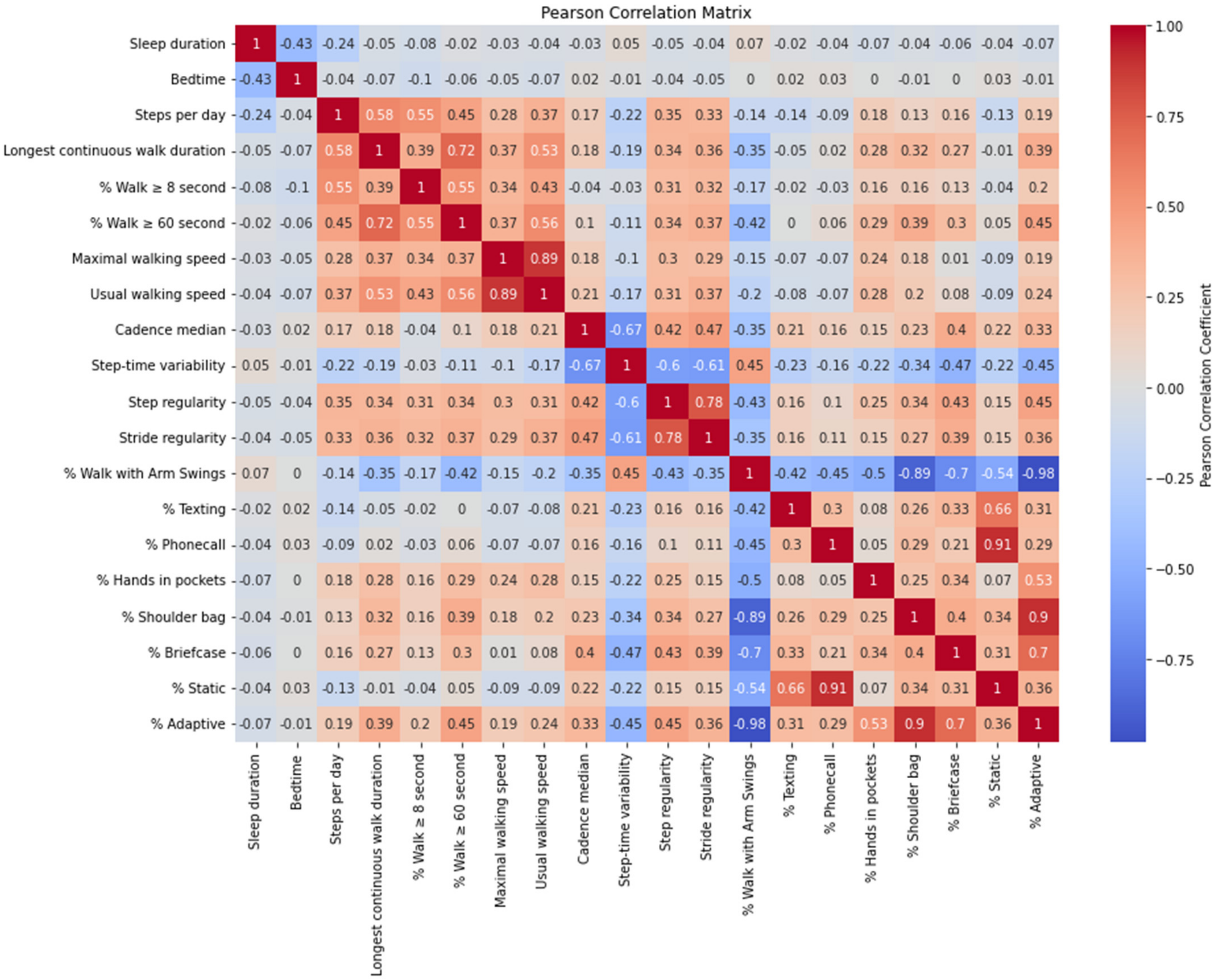

Supplementary Figure S4. Pearson's correlation coefficients among the digital biomarkers. (n = 73,438)
